# Evaluation of oxidative stress in ill neonatal foals: a preliminary study

**DOI:** 10.1093/jvimsj/aalag109

**Published:** 2026-06-12

**Authors:** Francesca Bindi, Dania Cingottini, Lucia de Marchi, Giulia Sala, Valentina Vitale, Micaela Sgorbini

**Affiliations:** Department of Veterinary Science, University of Pisa, San Piero a Grado, Pisa 56122, Italy; Department of Veterinary Science, University of Pisa, San Piero a Grado, Pisa 56122, Italy; Department of Veterinary Science, University of Pisa, San Piero a Grado, Pisa 56122, Italy; Department of Veterinary Science, University of Pisa, San Piero a Grado, Pisa 56122, Italy; Hospital Clínico Veterinario, Universidad CEU-Cardenal Herrera, CEU Universities, Alfara del Patriarca, Valencia 46115, Spain; Department of Veterinary Science, University of Pisa, San Piero a Grado, Pisa 56122, Italy

**Keywords:** ill foals, oxidative biomarkers, redox status, sepsis

## Abstract

**Background:**

Oxidative stress both contributes to and results from sepsis pathophysiology, but its role in ill foals remains poorly characterized.

**Hypothesis/Objectives:**

Ill foals will exhibit decreased antioxidant defense activity and increased oxidative products compared with healthy foals, and these changes will be associated with higher sepsis scores, lower survival scores, and non-survival.

**Animals:**

Twenty-six foals < 14 days old: 16 hospitalized foals (11 sick non-septic, 5 septic; median age, 1.5 days; range 1-13) and 10 healthy Standardbred foals (2 days old). Nine of 16 ill foals survived to discharge.

**Methods:**

Prospective multicenter cohort study. Ill foals were sampled at admission and every 24 h; healthy foals were sampled once at 48 h. Plasma paraoxonase (POase), arylesterase (AREase), superoxide dismutase (SOD), butyrylcholinesterase (BTChE), glutathione S-transferase (GST), glutathione peroxidase (GPx), total antioxidant capacity (TAC), and lipid peroxidation (LPO) were measured. Associations with sepsis score, survival score, and outcome were evaluated using generalized linear mixed models.

**Results:**

At admission, ill foals had lower SOD, GPx, BTChE, and GST and higher LPO compared with healthy foals (*P* ≤ .01). Non-survivors had lower POase (*P* = .01), SOD (*P* = .04), BTChE (*P* < .001), GST (*P* = .04), and GPx (*P* = .01) and higher LPO (*P* = .01). In mixed models, SOD, BTChE, and TAC were associated with both sepsis and survival score (*P* < .001). Arylesterase and POase were associated with sepsis score (*P* < .001).

**Conclusions and clinical importance:**

Ill neonatal foals had decreased antioxidant activity and increased oxidative products. Some biomarkers were associated with clinical scores and outcome, supporting further investigation of their potential clinical relevance.

## Introduction

Neonatal sepsis in foals is a common, life-threatening condition.^1^ Although sepsis activates immune responses needed for pathogen control, dysregulated inflammatory and oxidative pathways can cause tissue injury.^2^^,^^3^ Endogenous anti-inflammatory and antioxidant defenses are often overwhelmed, resulting in systemic inflammatory response syndrome (SIRS) and a redox imbalance that favors oxidative processes.^3^^,^^4^

Oxidative stress develops when reactive oxygen species (ROS) production exceeds antioxidant capacity, resulting in oxidative modification of lipids, proteins, and nucleic acids and promoting apoptosis and necrosis.^5^^,^^6^ Several biomarkers have been used to characterize oxidative status. Paraoxonase (POase), a negative acute phase protein, decreases during inflammation because of altered high-density lipoprotein (HDL) composition and decreased hepatic expression.^7^ Its activity can be evaluated through POase and arylesterase (AREase) activities.^8^ Lipid peroxidation (LPO) is widely used as an indicator of oxidative damage, because polyunsaturated fatty acids are highly susceptible to peroxidative injury.^9^ Antioxidant defenses are also central to limiting oxidative injury. Superoxide dismutase (SOD) converts superoxide radicals to hydrogen peroxide, which is subsequently metabolized by glutathione peroxidase (GPx). Glutathione S-transferase (GST) catalyzes the conjugation of glutathione to detoxifying xenobiotics and mitigates oxidative injury while supporting membrane integrity.^10^^,^^11^ Total antioxidant capacity (TAC) provides an integrated measure of overall antioxidant status. Butyrylcholinesterase (BTChE), an enzyme that hydrolyzes butyrylcholine and other esters, has been implicated in the inflammatory response via the cholinergic anti-inflammatory pathway.^12–18^

In septic human neonates, increased production of ROS has been reported, reflected by increased oxidative products and decreased antioxidant activity.^3^^,^^4^^,^^19^

Despite extensive research in human neonates, studies investigating oxidative injury in septic foals remain limited. A recent study provided important insight into redox imbalance in septic and critically ill foals by evaluating a broad panel of oxidative stress and antioxidant biomarkers.^20^ The authors reported that sick and septic neonatal foals had significantly increased markers of oxidative damage and lower concentrations of key antioxidant defenses, compared with healthy controls. These alterations also were associated with poor clinical outcomes, emphasizing the potential prognostic relevance of oxidative markers in the equine neonate.^20^

Our study aimed to evaluate oxidative stress and oxidative injury biomarkers in neonatal foals and to compare their activities among healthy, sick non-septic (SNS), and sick septic (SS) groups, as well as among healthy likely survivors (LS) and likely non-survivors (LNS). In addition, we aimed to assess the associations among biomarker activities, sepsis score, survival score, and clinical outcome.

## Materials and methods

### Animals

Sixteen foals admitted to the Veterinary Teaching Hospital of the University of Pisa and Cardinal Herrera University were enrolled in the study. All foals were < 15 days of age. Ill foals with a modified sepsis score (MSS) < 12 were classified as SNS, whereas those with MSS ≥ 12 were classified as SS.^21^ Based on survival score, ill foals were further categorized as LS when the survival score was ≥ 6 and as LNS when the score was < 6.^22^ Clinical outcome was defined as survivor or non-survivor at discharge (spontaneously died or euthanized because of worsening clinical signs). Ten foals born at a private breeding farm were examined 48 h after birth. Foals were considered healthy if they had a normal physical examination, vital signs within reference intervals, and serum IgG concentration > 800 mg/dL.

### Study design

Our prospective, multicenter in vivo study was approved by the Ethics Committee on Animal Experimentation of the University of Pisa (protocol nr. 03/2023).

In ill foals, blood samples were collected at admission (T0) and subsequently at 24-h intervals until discharge or death, for a maximum of 96 h (T24, T48, T72, and T96). Samples were collected in lithium-heparinized tubes and centrifuged within 30 min at 5000 × *g* for 10 min. Plasma then was harvested, and stored at −80°C.

In healthy foals, blood was collected once, 48 h after birth, during assessment of passive transfer of immunity, and a portion of the blood was processed and stored in the same manner as samples obtained from ill foals.

### Measurement of markers of oxidative stress and antioxidant activity

All laboratory assays used in the study (AREase, POase, SOD, BTChE, GST, GPx, TAC, and LPO) were performed using a spectrophotometric method with a Synergy HT Absorbance Microplate Reader (BioTek Co., USA). Please see Supplementary Table S1 in Supplementary Information for complete methods and assay details.

### Statistical analysis

Data were analyzed using IBM SPSS Statistics (IBM Corp., Armonk, NY, USA). Normality was assessed with the Shapiro–Wilk test. Group comparisons used the Mann–Whitney *U* test (healthy vs ill; survivors vs non-survivors) and the Kruskal–Wallis test with Bonferroni post hoc correction (healthy, SNS, SS; healthy, LS, LNS). Associations among biomarkers, sepsis score, survival score, and outcome in diseased foals were evaluated using generalized linear mixed models (GLMMs) with an inverse Gaussian distribution. Statistical significance was set at *P* < .05. Please see Supplementary Information for complete description of statistical analysis.

## Results

The study included 16 ill foals ranging in age from 1 to 13 (median age 1.5) days at admission. Seven of 16 were fillies (43.8%) and 9 were colts (56.2%). Five of 16 (31.3%) were American breeds (Quarter Horse and Appaloosa), 4/16 (25%) were Warmbloods, 3/16 (18.8%) were Standardbreds, 2/16 (12.5%) were Andalusian, 1/16 (6.3%) were ponies, and 1/16 (6.3%) were mixed breed. Ten healthy Standardbred foals, all 2 days old, also were included. Of these, 6/10 (60%) were colts and 4/10 (40%) were fillies. At admission, based on the MSS, 11/16 (68.8%) foals were SNS, and 5/16 (31.3%) were SS. According to the survival scores, 11/16 (68.8%) were LS, and 5/16 (31.3%) were LNS. Regarding outcomes, 9/16 (56.3%) foals survived, whereas 7/16 (43.8%) did not survive. Table S2 summarizes the age, diagnosis, sepsis score, survival score, and outcomes of the 16 foals included in the study.

Significant differences in POase activity were observed among groups. At T0, healthy foals had higher POase activity compared with SS foals (*P* = .01). Similarly, foals that survived had higher POase activity than non-survivors (*P* = .01). At T24, POase activity remained higher in foals that survived compared with non-survivors (*P* = .01). No other differences in POase were detected between healthy and ill foals or among LS and LNS groups at any time point.

The SOD activity was significantly higher in healthy foals compared with several ill groups. At T0, healthy foals had higher SOD activity than ill foals (*P* < .05). This difference remained significant at T24 (*P* < .01) and T48 (*P* < .01). Healthy foals also had higher SOD activity than SS (*P* = .01) and LNS foals (*P* = .01). The difference persisted at T48 between healthy and SS foals (*P* = .01). Foals that survived also had higher SOD activity than non-survivors at T0 (*P* = .04) and higher activity than LNS foals at T48 (*P* = .01).

At T0, BTChE activity was significantly lower in ill compared with healthy foals (*P* < .01), and foals that survived had higher BTChE activity than non-survivors (*P* < .001). No significant differences were found among other groups or time points.

Healthy foals had significantly higher GST activity than ill foals at T0 (*P* < .001), T24 (*P* < .001), and T48 (*P* < .001). At the same time point, foals that survived also had higher GST activity than non-survivors (*P* = .04). No other significant differences were detected across groups.

The GPx activity showed consistent differences across multiple comparisons. At T0, healthy foals had higher GPx activity than ill (*P* = .001), SNS and SS foals (both *P* = .01), and LS foals (*P* = .01). This difference persisted at T24, with higher GPx activity in healthy compared with ill (*P* < .001), SNS and SS foals (both *P* = .002), and both LS and LNS foals (*P* = .003). At T48, GPx activity remained higher in healthy than in ill (*P* < .01) and SS foals (*P* = .03), and foals that survived had higher GPx activity than non-survivors (*P* = .01).

At T0, LPO activity was significantly lower in healthy than in SS (*P* = .01) and LNS foals (*P* = .02). In the survival comparison, foals that survived also had lower LPO activity than non-survivors (*P* = .01). No significant differences in LPO activity were detected between healthy and ill foals at any time point.

No significant differences were observed among groups at any time point in AREase and TAC activities.

Table 1 summarizes the median values (IQR) of oxidative stress and antioxidant defense biomarkers in healthy foals (single sample 48 h after birth) and in ill foals at T0-T96.

**Table 1:** Median [IQR] values of oxidative stress biomarkers and antioxidant parameters in healthy and ill foals.

**Biomarker**	**Healthy**	**Ill T0**	**Ill T24**	**Ill T48**	**Ill T72**	**Ill T96**
**POase (μmol/mL/min)**	32.34 [21.78-41.56]	19.66 [17.02-30.27]	18.53 [14.86-29.28]	17.28 [14.39-38.03]	31.21 [15.97-37.26]	35.43 [21.94-64.84]
**AREase (kU/L)**	286.91 [168.90-519.72]	202.00 [140.03-262.22]	209.10 [178.93-271.11]	268.23 [122.28-398.33]	278.13 [181.67-551.66]	306.11 [187.28-450.56]
**LPO (MDA/μL)**	10.15 [9.74-11.47]	14.43 [11.29-17.31]	13.76 [7.78-18.27]	9.37 [6.60-23.17]	7.87 [5.46-18.42]	6.96 [5.28-15.47]
**SOD (U/mL)**	2.56 [2.21-2.80]	2.02 [1.26-2.55]	2.01 [0.94-2.49]	1.43 [0.65-2.35]	2.09 [0.87-2.42]	1.51 [1.02-1.98]
**BTChE (μmol/mL/min)**	16.72 [14.45-20.61]	16.08 [11.63-22.71]	16.10 [9.79-18.83]	16.84 [12.16-27.19]	12.33 [11.47-17.29]	17.79 [11.96-27.16]
**TAC (μmol/mL)**	4.00 [3.50-9.00]	5.75 [4.00-9.75]	5.00 [3.00-7.75]	5.50 [3.00-7.50]	5.00 [3.00-8.25]	6.50 [4.50-14.00]
**GST (U/mg protein)**	58.86 [24.28-80.06]	46.50 [25.52-74.67]	18.29 [11.59-30.96]	28.44 [19.35-49.13]	35.83 [17.03-45.05]	36.42 [24.25-58.18]
**GPx (U/mg protein)**	1.36 [0.62-1.52]	0.14 [0.03-0.79]	0.05 [0.03-0.66]	0.21 [0.02-0.73]	0.18 [0.02-0.65]	0.68 [0.37-0.91]
**Protein (g/L)**	112.80 [100.52-123.70]	99.79 [83.49-127.07]	106.31 [95.42-132.56]	112.87 [99.28-143.35]	127.88 [105.12-141.63]	136.24 [113.10-156.19]

Abbreviations: AREase = arylesterase; BTChE = butyrylcholinesterase; GPx = glutathione peroxidase; GST = glutathione S-transferase; LPO = lipid peroxidation; MDA = malondialdehyde; POase = paraoxonase; SOD = superoxide dismutase; TAC = total antioxidant capacity.

The GLMMs, including repeated measures over time and performed on the 16 ill foals showed significant associations for several biomarkers.

The SOD activity was significantly associated with both sepsis score (*P* < .001) and survival score (*P* < .001).

Compared with the reference sepsis score category 16, the following were significant:

- Sepsis score 0: β = 2.957 (95% CI, 2.241-3.672), *P* < .001
- Sepsis score 7: β = 2.963 (95% CI, 2.262-3.665), *P* < .001
- Sepsis score 8: β = 1.515 (95% CI, 1.011-2.019), *P* < .001
- Sepsis score 10: β = 1.596 (95% CI, 1.059-2.133), *P* < .001

Compared with the reference survival score category 3:

- Survival score 6: β = 2.681 (95% CI, 2.062-3.301), *P* < .001
- Survival score 7: β = 2.531 (95% CI, 1.915-3.148), *P* < .001

Butyrylcholinesterase was significantly associated with sepsis score (*P* < .001) and survival score (*P* < .001).

Compared with sepsis score 16:

- Sepsis score 0: β = −8.905 (95% CI, −15.337 to −2.472), *P* = .01

Compared with survival score 3:

- Survival score 6: β = 7.332 (95% CI, 3.114-11.551), *P* = .001
- Survival score 7: β = 8.615 (95% CI, 4.321-12.909), *P* < .001

Total antioxidant capacity showed strong associations with sepsis score (*P* < .001) and survival score (*P* < .001).

Compared with sepsis score 16:

- Sepsis score 0: β = 9.713 (95% CI, 8.345-11.082), *P* < .001
- Sepsis score 8: β = −3.970 (95% CI, −5.004 to −2.936), *P* < .001
- Sepsis score 13: β = −4.459 (95% CI, −5.494 to −3.425), *P* < .001

Compared with survival score 3:

- Survival score 6: β = 2.841 (95% CI, 1.553-4.129), *P* < .001
- Survival score 7: β = 3.102 (95% CI, 1.811-4.392), *P* < .001

Arylesterase activity was significantly associated with sepsis score (*P* < .001), but not with survival score (*P* = .3).

Compared with sepsis score 16:

- Sepsis score 0: β = 384.525 (95% CI, 274.111-494.939), *P* < .001
- Sepsis score 7: β = 205.288 (95% CI, 102.064-308.511), *P* < .001
- Sepsis score 13: β = 94.507 (95% CI, 10.471-178.543), *P* = .03

No survival score coefficients were significant.

Paraoxonase activity was significantly associated with sepsis score (*P* < .001), but not with survival score (*P* = .07).

Compared with sepsis score 16:

- Sepsis score 0: β = 17.371 (95% CI, 6.553-28.188), *P* = .002
- Sepsis score 7: β = 17.448 (95% CI, 7.161-27.734), *P* = .001
- Sepsis score 8: β = 55.617 (95% CI, 47.622-63.613), *P* < .001

No survival score coefficients were significant.

Glutathione peroxidase, GST, and LPO showed no significant associations with survival score and no consistent significant effects relative to sepsis reference category. The full summary of GLMM fixed effects for oxidative stress biomarkers, including β estimates, 95% CI, and *P*-values is presented in Table S3.

## Discussion

We evaluated oxidative stress biomarkers and antioxidant enzymes in ill neonatal foals, comparing them with healthy foals and stratified results according to sepsis score, survival score, and outcome classifications. Overall, the findings suggest that subsets of ill foals showed evidence of oxidative imbalance, with decreased activities of several antioxidant-related enzymes and increased LPO in selected severity and outcome comparisons. The most consistent differences between healthy and ill foals were observed for SOD, GST, and GPx, whereas POase and LPO differed mainly in specific subgroup comparisons. Moreover, GLMM analysis identified significant associations of SOD, BTChE, TAC, AREase, and POase with sepsis or survival score categories or both, whereas GPx, GST, and LPO showed no consistent score-related effects.

A decrease in POase activity was detected in the SS group compared with healthy foals. In human patients and neonates affected by sepsis, a similar decrease has been reported as a consequence of oxidative stress.^23^^,^^24^ No studies have evaluated POase activity in foals, but in adult horses with experimentally induced endotoxemia, decreased POase activity has been observed as a secondary oxidative phenomenon associated with endotoxemia.^25^ Paraoxonase activity plays an important role in antioxidant defense by hydrolyzing lipid peroxides and limiting oxidative damage associated with ROS. During sepsis, excessive ROS production and systemic inflammation may lead to increased oxidative consumption of the enzyme and functional alterations of HDL, with which POase is associated.^7^ The decrease observed in SS foals in our study therefore supports the hypothesis that POase-related antioxidant mechanisms may be impaired during systemic inflammation in neonatal foals.

At admission and over the first 48 h of hospitalization, ill foals had lower SOD activity than healthy foals. In SS foals, lower SOD activity was evident at admission and remained significant at T48. A significant decrease in SOD activity has been reported in septic human patients compared with healthy controls.^26^ Similarly, foals with lower respiratory tract infections and sepsis exhibit decreased SOD activity compared with healthy control groups.^20^^,^^27^ The observed decrease in SOD activity potentially may result in inadequate protection of organs against ROS-mediated oxidative damage during sepsis.^28^

In our study, BTChE activity at T0 was lower in ill foals than in healthy foals, and foals that survived had higher BTChE activity than non-survivors. These findings are consistent with evidence from human medicine showing that decreased BTChE activity is associated with adverse outcomes in various inflammatory and critical conditions such as chronic obstructive pulmonary disease and coronavirus (COVID-19) pneumonia, cancer, and coronary artery disease.^14–18^ A recent review further highlighted that low BTChE activity correlates with systemic inflammation and increased mortality across multiple diseases, including sepsis and other systemic disorders, emphasizing its potential as a general biomarker of disease progression.^29^ Butyrylcholinesterase hydrolyzes choline esters and is increasingly recognized as a marker of systemic inflammatory status. During sepsis, reduced BTChE activity may reflect decreased hepatic synthesis and increased enzyme consumption in the presence of systemic inflammation and oxidative stress. In addition, alterations in cholinesterase activity may influence acetylcholine-mediated signaling involved in the cholinergic anti-inflammatory pathway, which modulates the host inflammatory response. Therefore, decreased BTChE activity in critically ill foals may indicate impaired metabolic and inflammatory regulation during systemic disease.^12–18^

Ill foals had lower GST activity than healthy foals during the first 48 h of hospitalization. Glutathione peroxidase activity was also lower in ill foals and in SS foals compared with healthy foals over this period. Glutathione S-transferase and GPx are key components of the glutathione-dependent antioxidant defense system. Glutathione S-transferase catalyzes the conjugation of reduced glutathione to electrophilic compounds and toxic oxidation products, facilitating their detoxification and excretion, whereas GPx reduces hydrogen peroxide and lipid hydroperoxides to less reactive molecules, thereby limiting oxidative damage to cellular components.^10^^,^^11^ Decreased activity of glutathione has been described in several species during illness. In humans, low glutathione activity has been described during sepsis.^30–32^ Similar findings have been reported in sick foals, where significantly lower glutathione concentrations were detected compared with healthy controls.^20^^,^^33^ The lower GPx and GST activities in ill foals may result from excessive oxidative stress leading to glutathione depletion and decreased enzyme activity because of limited substrate availability and impaired antioxidant regeneration.^30^^,^^31^

Higher LPO activity was detected in SS foals compared with healthy foals at T0. In addition, LPO activity was higher in LNS foals and non-survivors than in their respective comparison groups at admission. In septic human patients and neonates, higher malondialdehyde (MDA) concentrations have been reported.^34^^,^^35^ Similarly, higher MDA concentrations have been described in ill foals compared with control groups, and the mean MDA concentrations were higher in more severe illness groups.^20^ The increased LPO activity observed in these foals suggests that oxidative stress, particularly LPO, may play a role in the pathophysiology of illness and sepsis in neonatal foals.^20^^,^^36^

Significant differences in biomarkers also were observed among healthy, LS, and LNS foals, but no significant differences were detected between LS and LNS foals. Notably, higher LPO activity along with lower SOD and GPx activities were detected in LNS foals compared with healthy foals. To date, oxidative stress biomarkers have not been evaluated in association with survival scores in neonatal foals. The pattern observed in LNS foals, including higher LPO and lower antioxidant enzyme activity in selected comparisons with healthy foals, may indicate impaired antioxidant defense in foals with worse predicted prognosis. However, because no significant differences were detected between LS and LNS foals, this interpretation should be considered preliminary. The absence of significant differences between LS and LNS foals in our study may indicate that both groups experienced a comparable degree of oxidative and inflammatory stress, regardless of the predicted survival outcome. Alternatively, the lack of distinction could be attributed to the limited number of foals in the LS and LNS groups, which may have decreased the statistical power to detect subtle differences.

Significant differences in biomarkers were observed between the survivor and non-survivor groups. Compared with survivors, non-survivor foals had lower POase, SOD, BTChE, and GST activities and higher LPO activity at admission, whereas lower GPx activity in non-survivors became evident at T48. In septic human patients, SOD activity was significantly lower in non-survivor patients than in surviving patients.^23^^,^^37^ Moreover, lower glutathione activities have been reported in non-survivor septic human patients compared with control groups.^38–41^ In foals, SOD and glutathione activities have been reported to be lower in non-surviving individuals.^20^ In our study, the findings support the hypothesis that increased oxidative stress and impaired antioxidant response during sepsis might be associated with an unfavorable outcome.^32^^,^^42^

The GLMM analysis further demonstrated that several antioxidant enzymes were associated with clinical severity. Superoxide dismutase and BTChE activities were significantly associated with both sepsis and survival scores, suggesting that impaired antioxidant defense and systemic inflammatory responses may contribute to worse clinical status in septic foals. Similar alterations in antioxidant enzymes and cholinesterase activity have been reported in neonatal foals and in human patients with sepsis and other critical illnesses.^20^^,^^29^ In contrast, POase and AREase activities were associated only with sepsis score, indicating that POase-related antioxidant mechanisms may reflect disease severity.^25^ Total antioxidant capacity also showed significant associations with both clinical scores despite the absence of differences in the pairwise comparisons, suggesting that longitudinal modeling may detect subtle changes in the global antioxidant capacity during illness. Conversely, GPx, GST, and LPO were not significantly associated with the clinical scores, indicating that these markers may be less closely related to disease severity when temporal changes are considered.

The main limitations of our study include a relatively small sample size and the heterogeneity of diseases within the ill group. An additional important limitation relates to the study design, in which healthy control foals were sampled only once at a single time point, whereas hospitalized foals were evaluated longitudinally. This difference in sampling design limits direct comparisons among groups over time and may introduce potential age-related confounding effects. The healthy control foals constituted a convenience sample of Standardbred neonates undergoing routine clinical evaluation of passive transfer of immunity after birth. In our clinical setting, blood collection from healthy neonatal foals is ethically justified only when performed for clinically indicated procedures. Therefore, repeated or age-stratified sampling of healthy controls solely for research purposes was not feasible. Consequently, the control group consisted of Standardbred neonates of similar age, whereas the hospitalized foals represented a mixed-breed population sampled across a broader age range. Age-related physiological variation in oxidative stress and antioxidant biomarkers during early life therefore may have influenced the observed differences among groups.^43^ In addition, sepsis status was defined based on the sepsis score, because blood cultures were not performed in all foals in the study and therefore were not analyzed. This factor may have limited the ability to confirm septic status microbiologically. Although a wide variety of oxidative stress and antioxidant biomarkers were measured, additional antioxidants, such as ascorbic acid, vitamin E, and selenium, as well as other biomarkers of oxidative injury, could have provided a more comprehensive assessment of redox status in neonatal foals. The inclusion of markers associated with reactive nitrogen species also might have helped further clarify the interactions between oxidative and nitrosative pathways in SS foals. These factors should be considered when interpreting our conclusions.

Collectively, our findings emphasize the importance of oxidative stress as a pathophysiologic component of sepsis in foals. Our findings reinforce the evidence that ill neonatal foals may experience oxidative imbalance similar to that reported in septic human neonates, reflected by altered oxidative stress biomarkers and decreased antioxidant enzyme activities.

## Supplementary Material

Supplementary_tables_ready_to_publish_aalag109

## Off-label antimicrobial declaration

The authors declare no off-label use of antimicrobials.

## Institutional animal care and use committee or other approval declaration

Approved by the Ethics Committee on Animal Experimentation of the University of Pisa (Protocol No. 03/2023). Owners’ written consent for the plasma collection was obtained for all the foals included in this study.

## Abbreviations

AREase: arylesterase
BTChE: butyrylcholinesterase
GLMMs: generalized linear mixed models
GPx: glutathione peroxidase
GST: glutathione S-transferase
HDL: high-density lipoprotein
LNS: likely non-survivors
LPO: lipid peroxidation
LS: likely survivors
MDA: malondialdehyde
MSS: modified sepsis score
POase: paraoxonase
ROS: reactive oxygen species
SIRS: systemic inflammatory response syndrome
SNS: sick non-septic
SOD: superoxide dismutase
SS: sick septic
TAC: total antioxidant capacity
VTH: veterinary teaching hospital
