# Supplementary material for: Evaluation of oxidative stress in ill neonatal foals: a preliminary study
Source: J Vet Intern Med. 2026 Jun 12;40(3):aalag109. doi: 10.1093/jvimsj/aalag109 (PMC13262654; doi:10.1093/jvimsj/aalag109)
Supplement: Supplementary_tables_ready_to_publish_aalag109 [file supplementary_tables_ready_to_publish_aalag109.docx]

**Supplementary table 1.** Analytical performance and validation parameters of spectrophotometric assays used for oxidative stress biomarkers. **Abbreviations:** AREase, Arylesterase; BTChE, Butyrylcholinesterase; CV, coefficient of variation; GPx, Glutathione peroxidase; GST, Glutathione S-transferase; LPO, Lipid peroxidation; MDA, malondialdehyde; POase, Paraoxonase; R², coefficient of determination; SOD, Superoxide dismutase; TAC, Total antioxidant capacity.

| Biomarker | Calibration range | Curve model | R² | Intra-assay CV (%) | Inter-assay CV (%) | Reference control material |
| --- | --- | --- | --- | --- | --- | --- |
| POase | 0.4 – 45 µmol p-nitrophenol/mL | Linear | 0.995 | 4.5 | 6.7 | In-house pooled foal plasma quality control (aliquoted and stored at −80 °C) |
| AREase | 4 – 28 mM acetic acid equivalents | Linear | 0.993 | 5.3 | 7.0 | In-house pooled foal plasma quality control (aliquoted and stored at −80 °C) |
| LPO | 0.4 – 10 µmol MDA/mL | Linear | 0.990 | 6.2 | 8.4 | MDA calibration standard generated from 1,1,3,3-tetramethoxypropane (TMP; Cayman |
| SOD | 0.04 – 18 U/mL | Non-linear (4PL) | 0.988 | 3.7 | 6.4 | Cu/Zn-SOD (bovine erythrocytes) quality control standard (Sigma-Aldrich) |
| BTChE | 4 – 180 µmol substrate hydrolysed/min | Linear | 0.997 | 4.0 | 5.8 | Purified butyrylcholinesterase enzyme standard (Sigma-Aldrich) |
| TAC | 120 – 1800 µmol Fe²⁺/L | Linear | 0.996 | 3.6 | 5.1 | Ferrous sulfate (Fe²⁺) FRAP reference standard (Cayman) |
| GST | 0.15 – 14 U/mL | Linear | 0.994 | 5.5 | 7.2 | Glutathione S-transferase enzymatic standard (Sigma-Aldrich) |
| GPx | 0.04 – 9 U/mL | Non-linear (4PL) | 0.991 | 4.7 | 6.5 | Glutathione peroxidase control standard (Randox) |
| Protein | 0.04 – 1.8 mg/mL | Linear | 0.998 | 2.6 | 3.9 | Bovine serum albumin certified reference standard (Thermo Fisher) |

**Supplementary table 2.** Details of foals enrolled in the study, including age, diagnosis, sepsis score, surviva score, outcome.

| Patient | Age (days) | Diagnosis | Sepsis score | Survival score | Outcome |
| --- | --- | --- | --- | --- | --- |
| 1 | 7 | Meconium impaction | 8 | 4 | Survived |
| 2 | 3 | Interstitial pneumonia | 15 | 7 | Survived |
| 3 | 3 | Meconium impaction and bladder rupture | 3 | 7 | Dead |
| 4 | 1 | Neonatal isoerythrolysis and septic arthritis | 7 | 6 | Survived |
| 5 | 1 | Neonatal isoerythrolysis and septic arthritis | 19 | 6 | Dead |
| 6 | 13 | Rhodococcosis, polyarthritis/polysynovitis | 3 | 5 | Survived |
| 7 | 2 | Aspiration pneumonia | 16 | 3 | Dead |
| 8 | 1 | Prematurity | 19 | 1 | Dead |
| 9 | 1 | Dysphagia and pneumonia | 12 | 4 | Survived |
| 10 | 3 | Pneumonia, hepatic encephalopathy | 7 | 6 | Survived |
| 11 | 6 | Septic arthritis | 10 | 6 | Survived |
| 12 | 1 | Aspiration pneumonia | 10 | 7 | Survived |
| 13 | 1 | Dysphagia | 0 | 6 | Survived |
| 14 | 3 | Meconium impaction | 13 | 4 | Dead |
| 15 | 1 | Pneumonia | 10 | 6 | Survived |
| 16 | 1 | Shock | 9 | 6 | Dead |

**Supplementary table 3. Fixed effects from generalized linear mixed models (GLMM) evaluating the association of sepsis score, survival score, and outcome with oxidative stress biomarkers in ill foals (n = 16). Reference categories: Sepsis score = 16, Survival score = 3, Outcome = non-survivor. Only statistically significant estimates are shown unless otherwise indicated. Abbreviations:** AREase, Arylesterase; BTChE, Butyrylcholinesterase; GPx, Glutathione peroxidase; GST, Glutathione S-transferase; LPO, Lipid peroxidation; MDA, malondialdehyde; POase, Paraoxonase; SOD, Superoxide dismutase; TAC, Total antioxidant capacity.

| **Biomarker** | **Predictor** | **Category** | **β** | **95% CI** | **p-value** |
| --- | --- | --- | --- | --- | --- |
| **POase (µmol/mL/min)** | Sepsis score | 0 | 17.371 | 6.553–28.188 | .002 |
|  |  | 7 | 17.448 | 7.161–27.734 | .001 |
|  |  | 8 | 55.617 | 47.622–63.613 | <.001 |
|  | Survival score | – | – | – | >.05 |
|  | Outcome | Survived | – | – | >.05 |
| **AREase (kU/L)** | Sepsis score | 0 | 384.525 | 274.111–494.939 | <.001 |
|  |  | 7 | 205.288 | 102.064–308.511 | <.001 |
|  |  | 13 | 94.507 | 10.471–178.543 | .028 |
|  | Survival score | – | – | – | >.05 |
|  | Outcome | Survived | – | – | >.05 |
| **SOD (U/mL)** | Sepsis score | 0 | 2.957 | 2.241–3.672 | <.001 |
|  |  | 7 | 2.963 | 2.262–3.665 | <.001 |
|  |  | 8 | 1.515 | 1.011–2.019 | <.001 |
|  |  | 10 | 1.596 | 1.059–2.133 | <.001 |
|  | Survival score | 6 | 2.681 | 2.062–3.301 | <.001 |
|  |  | 7 | 2.531 | 1.915–3.148 | <.001 |
|  | Outcome | Survived | – | – | >.05 |
| **BTChE (µmol/mL/min)** | Sepsis score | 0 | −8.905 | −15.337 to −2.472 | .008 |
|  | Survival score | 6 | 7.332 | 3.114–11.551 | .001 |
|  |  | 7 | 8.615 | 4.321–12.909 | <.001 |
|  | Outcome | Survived | - | – | >.05 |
| **TAC (µmol/mL)** | Sepsis score | 0 | 9.713 | 8.345–11.082 | <.001 |
|  |  | 8 | −3.970 | −5.004 to −2.936 | <.001 |
|  |  | 13 | −4.459 | −5.494 to −3.425 | <.001 |
|  | Survival score | 6 | 2.841 | 1.553–4.129 | <.001 |
|  |  | 7 | 3.102 | 1.811–4.392 | <.001 |
|  | Outcome | Survived | – | – | >.05 |
| **LPO (MDA/μL)** | All predictors | – | – | – | >.05 |
| **GPx (U/mg protein)** | All predictors | – | – | – | >.05 |
| **GST (U/mg protein)** | All predictors | – | – | – | >.05 |
